# Meta-analyses in psychology often overestimate evidence for and size of effects

**DOI:** 10.1098/rsos.230224

**Published:** 2023-07-05

**Authors:** František Bartoš, Maximilian Maier, David R. Shanks, T. D. Stanley, Martina Sladekova, Eric-Jan Wagenmakers

**Affiliations:** ^1^ Department of Psychological Methods, University of Amsterdam, Amsterdam, The Netherlands; ^2^ Department of Experimental Psychology, University College London, London, UK; ^3^ Deakin Laboratory for the Meta-Analysis of Research (DeLMAR), Deakin University, Burwood, Australia; ^4^ Department of Economics, School of Business, Deakin University, Burwood, Australia; ^5^ School of Psychology, University of Sussex, Brighton, UK

**Keywords:** publication bias, RoBMA, meta-analysis, model-averaging, model-selection, Bayesian inference

## Abstract

Adjusting for publication bias is essential when drawing meta-analytic inferences. However, most methods that adjust for publication bias do not perform well across a range of research conditions, such as the degree of heterogeneity in effect sizes across studies. Sladekova *et al*. 2022 (Estimating the change in meta-analytic effect size estimates after the application of publication bias adjustment methods. *Psychol. Methods*) tried to circumvent this complication by selecting the methods that are most appropriate for a given set of conditions, and concluded that publication bias on average causes only minimal over-estimation of effect sizes in psychology. However, this approach suffers from a ‘Catch-22’ problem—to know the underlying research conditions, one needs to have adjusted for publication bias correctly, but to correctly adjust for publication bias, one needs to know the underlying research conditions. To alleviate this problem, we conduct an alternative analysis, robust Bayesian meta-analysis (RoBMA), which is not based on *model-selection* but on *model-averaging*. In RoBMA, models that predict the observed results better are given correspondingly larger weights. A RoBMA reanalysis of Sladekova *et al*.’s dataset reveals that more than 60% of meta-analyses in psychology notably overestimate the evidence for the presence of the meta-analytic effect and more than 50% overestimate its magnitude.

## 1. Introduction

Meta-analysis is widely regarded as the best way to combine and summarize seemingly conflicting evidence across a set of primary studies. However, publication bias—the preferential publishing of statistically significant studies—often causes meta-analyses to overestimate mean effect sizes [1–3]. Therefore, a key question concerns the extent to which meta-analytic estimates represent reliable indicators even when publication bias is left unaccounted for. To address this question, Sladekova, Webb and Field (2022; henceforth SWF) compiled an extensive dataset of 433 meta-analyses from the field of psychology and assessed the typical overestimation of effect sizes using methodologically advanced techniques and a model-selection procedure recently developed by Carter *et al.* [4]. SWF concluded that on average, effect size estimates were only marginally lower after accounting for publication bias. The most aggressive average adjustment was provided by precision effect test (PET) models, Δ*r* = −0.032, 95% CI [−0.055,−0.009]); moreover, meta-analyses comprising few studies often exhibited an anomalous upward adjustment.

In their analyses, SWF specified four plausible data-generating processes and selected the best estimator for each based on the findings of a simulation study by Carter *et al.* [4]. As different publication bias adjustment methods are generally found to perform well under different conditions, Carter *et al.* [4] provided code that allows researchers to select the most suitable publication bias correction method based on specific assumed research conditions, such as the true degree of heterogeneity in the effect sizes included in the meta-analysis. This approach presents a substantial improvement over the common practice of applying bias correction methods with little regard to the observed meta-analytic conditions. In theory, the meta-analytic conditions in terms of heterogeneity and *p*-hacking could be derived from external sources or knowledge of the research area. However, we believe that in empirical practice this is nigh impossible to execute as intended as it is difficult—especially in the case of a meta-meta analysis—to accurately estimate the size of these characteristics from external knowledge. This only leaves researchers with a second option: estimating them from the data. Unfortunately, research characteristics (i.e. the true effect size and degree of heterogeneity, and the degree and type of publication bias) cannot be accurately estimated unless one first adjusts for publication bias. Alternatively, specifying multiple conditions might result in different estimates, leaving the analyst with incompatible conclusions. Therefore, the approach by Carter *et al.* [4], as employed by SWF, creates a Catch-22 problem [5]: to correctly adjust for publication bias, one needs to know the underlying research conditions; however, in order to know the underlying research conditions, one needs to have adjusted correctly for publication bias.^1^

A second challenge for the ‘select-the-best-estimator’ approach is that the Carter *et al.* [4] simulation is based on specific assumptions about the data generating process. As with all simulations, the question is how well the data generating process actually corresponds to publication bias as it operates in the real world [8]. In their discussion, SWF point out that an alternative solution is provided by Bayesian model-averaging [9,10]. Bayesian model-averaging [e.g. robust Bayesian meta-analysis or RoBMA; 11–13] simultaneously considers an entire ensemble of models for publication selection and potential research conditions. The data then guides the inference to be based most strongly on those models that best predict the observed research results. In this way, Bayesian model-averaging of publication bias models alleviates the Catch-22 problem outlined above. SWF discuss how RoBMA would be a good alternative approach; here, we follow SWF’s suggestion and re-analyse their dataset with RoBMA. To preview, a very different (and, we argue, more credible) conclusion emerges from this re-analysis.

A third challenge for the ‘select-the-best-estimator’ approach is that investigations based on empirical data show that the specific correction methods employed by SWF do not adjust for publication bias sufficiently. In particular, Kvarven *et al.* [14] compared estimates from publication bias-adjusted meta-analyses to Registered Replication Reports on the same topic [15,16]. Registered Reports are a publication format in which a submitted manuscript receives peer review and ‘in principle’ acceptance based on the introduction and methods section alone. Hence the journal commits itself to publishing the report independent of the outcome, as long as the data pass pre-specified outcome-neutral quality checks. Therefore, Registered Reports are not affected by publication bias and can be considered the ‘gold standard’ of evidence. Consequently, a publication bias adjustment method that works well ought to produce an effect size estimate that is similar to the one from a Registered Report on the same topic. By comparing Registered Reports to associated meta-analyses, Kvarven *et al.* [14] showed that the publication bias correction methods employed in SWF lead to substantial overestimation of effect size and underestimation of the required correction (but see [17] for a criticism of this approach, which argues that the difference might partly be explained by genuine effect heterogeneity rather than publication bias). By contrast, Bartoš *et al.* [11] demonstrated that RoBMA generates estimates that are less biased and have considerably lower root mean square errors.

Finally, in their work SWF focus solely on the impact of publication bias adjustment on meta-analytic effect size. In practice, researchers also wish to know whether there is a genuine effect in the first place [18,19]. A Bayesian analysis allows us to quantify the evidence for a non-null effect and assess its posterior probability, while circumventing problems of frequentist significance testing [e.g.^20^,^21^].

In sum, by applying multiple models to the data simultaneously, RoBMA avoids the Catch-22 problem that plagues the ‘select-the-best-estimator’ approach. Moreover, RoBMA does not underadjust for publication bias [11], and offers a Bayesian way to quantify the extent to which publication bias inflates the evidence for the presence of an overall effect.

In the next sections, we apply RoBMA to the meta-analysis dataset compiled by SWF. The RoBMA re-analysis shows that many meta-analyses suffer from publication bias in the sense that both the effect size and the evidence for the presence of the effect are substantially overestimated (52.7% and 60.8%, respectively).

## 2. Method

### 2.1. The RoBMA model ensemble

Here, we describe how we employed the robust Bayesian model-averaging methodology. The remaining publication bias adjustment methods used in SWF are explained in more detail therein and in Carter *et al.* [4].

The complete RoBMA-PSMA model ensemble (as implemented in [11]; simply referred to below as RoBMA) employs models that can be categorized along three research dimensions: presence versus absence of the effect, heterogeneity across reported effects, and publication selection bias. Each of these hypotheses is assigned a prior model probability of 1/2, reflecting a position of equipoise. The individual models specified within the RoBMA ensemble then represent a combination of these research characteristics with prior model probabilities corresponding to the product of prior probabilities of each corresponding hypothesis. For models representing the presence of publication bias, the prior model probability is equally split among the various selection models and the PET and precision-effect estimate with standard errors (PET-PEESE), and then further split equally among the different selection models or between PET and PEESE. The complete RoBMA-PSMA ensemble consists of 36 different models.

The hypothesis that the effect is absent is represented by a point prior distribution on the effect size at *μ* = 0, and the hypothesis about the presence of the effect is represented by a standard normal prior distribution on Cohen’s *d* effect size, *μ* ∼ Normal(0, 1), representing a plausible range of effect sizes for psychology. We further offer an alternative analysis, which uses the Oosterwijk prior Student-*t*_+_ (*μ* = 0.35, *σ* = 0.10, *ν* = 3) on the effect size. This prior was elicited specifically for psychology and is not centred at zero but at effect sizes typical for the field (*d* = 0.35) [22]. The hypothesis that heterogeneity is absent is represented by a point prior distribution on the heterogeneity at 0, *τ* = 0, and the hypothesis about the presence of heterogeneity is represented by an inverse-gamma distribution, *τ* ∼ Inverse-Gamma (1, 0.15) (with scale and shape parameterization; corresponding to Cohen’s *d* effect sizes), based on empirical heterogeneity estimates from the field of psychology [23]. The hypothesis that publication bias is absent is instantiated by not applying any publication bias corrections, and the hypothesis about the presence of publication bias is instantiated by applying a set of six weight functions [2,24,25], and both the PET and PEESE models [26] to adjust for publication bias. The weight functions are specified as a combination of cut-offs on significant and marginally significant *p*-values, and the direction of the effect. The cumulative unit Dirichlet prior distributions enforce a decreasing relative prior probability with increasing *p*-values which further helps with the performance of selection models. The PET and PEESE models are specified as meta-regressions of the effect sizes on the standard errors or standard errors squared with truncated Cauchy distributions on the PET and PEESE regression coefficients, PET ∼ Cauchy_+_(0, 1), PEESE ∼ Cauchy_+_(0, 5), which enforce a positive relationship between standard errors and effect sizes. More details on the RoBMA specification are presented in Bartoš *et al.* [11].

The performance of RoBMA has been evaluated extensively in simulation studies as well as empirical comparisons. In particular, Bartoš *et al.* [11] reanalysed a large simulation study by Hong and Reed [27], which itself combined four different previous simulation environments comprising 1640 separate experimental conditions [4,26,28,29]. In these simulations, RoBMA outperformed other methods for publication bias correction in terms of bias and root mean squared error (RMSE). RoBMA was also evaluated empirically by comparing meta-analyses that are linked to Registered Replication Reports in Kvarven *et al.* [14]. As discussed above, comparing meta-analysis bias corrections to a ‘ground truth’ as revealed by Registered Reports allows us to evaluate whether a given correction sufficiently adjusts for likely publication bias. In the Kvarven *et al.* [14] comparison of meta-analyses and Registered Reports, RoBMA was shown to provide the best adjustment for publication bias when evaluated by average bias and/or root mean square error by Bartoš *et al.* [11]. Nonetheless, RoBMA and Bayesian model-averaging are only as good as the models incorporated in the ensemble. Since none of the meta-analytic models employed in RoBMA directly adjusts for *p*-hacking, RoBMA can exhibit downward bias in cases with strong *p*-hacking [11].

### 2.2. Dataset

This section gives a short summary of the dataset following Sladekova *et al.* [30]. Initially, a dataset comprising a random sample of a total of 169 meta-analyses published between 2008 and 2018 was selected. A study from this dataset was then included in the final dataset if (a) raw data were extractable, (b) the effect size was reported as a correlation coefficient *r* or sufficient information to transform the effect size was given and (c) information about the variance of primary studies’ estimates was included. Further, studies were excluded if (a) the study was a meta-meta-analysis or a meta-analysis using internal databases or (b) the original analyses failed to reproduce. Of the initial 169 articles, 52 were excluded because the data could not be obtained, 7 because they used incompatible effect sizes, 3 because they were internal meta-analyses and 2 because they were meta-meta-analyses. Of the remaining articles a further 22 datasets were excluded because the analyses failed to reproduce, 9 because the effect sizes could not be converted and 1 because it was a methodological report. The final sample included 433 datasets from 90 articles. For more details about the selection procedure, see [30, p. 6]. Here, we focus on the 406 estimates that SWF shared in their public OSF repository.

### 2.3. Effect size transformation

In contrast to SWF, we analysed the effect sizes using the Fisher *z* scale (and subsequently transformed the meta-analytic estimates back to the correlation scale for interpretation). We prefer the Fisher *z*-scale for two reasons. First, it is unbounded (i.e. not restricted to the [ −1, 1] interval) and the sampling distribution is approximately normal, which corresponds to the likelihoods used by meta-analytic models (this also prevents adjusted meta-analytic correlation estimates falling outside of [ −1, 1], which is anomalous).^2^ Second, the Fisher *z*-score and its standard error are by definition orthogonal, which is an important assumption for models adjusting for the relationship between effect sizes and standard errors such as PET-PEESE (this was not an issue in SWF as they used standard errors of Fisher’s *z* alongside the correlation effect sizes).

The use of Fisher’s *z*-scale results in slight differences in (a) selected methods for each condition (as the reduced range of correlation effect sizes limits the possible heterogeneity) and (b) effect size estimates of those selected methods. These differences, however, do not change the qualitative conclusions.

### 2.4. Effects of publication bias on evidence and effect size

We extended the SWF results by first assessing the extent to which publication bias inflates the evidence for the presence of an effect. Then, similarly to SWF, we also evaluated and compared the effect of publication bias on the meta-analytic estimates of the effect size.

To evaluate the change in evidence for the presence of the effect, we compared the posterior probability for the presence of the effect under RoBMA to the posterior probability for the presence of the effect under RoBMA after excluding the models that adjust for publication bias. The publication bias unadjusted version of RoBMA corresponds to a Bayesian model-averaged meta-analysis [BMA; e.g. 22,31,32]. For both RoBMA and BMA, the prior model probability for the presence of the effect is set to 1/2. Furthermore, we summarize the results as the change in the percentage of meta-analyses that provide at least moderate or strong evidence for either the null or alternative hypothesis based on the ‘rule of thumb’ Bayes factor categories that have been proposed to facilitate the interpretation of Bayes factors [i.e. BF > 3 is moderate evidence, and BF > 10 is strong evidence; 18,33].

To evaluate the change in the meta-analytic estimate of effect size, we compared the model-averaged posterior mean obtained from RoBMA to effect size estimates from two meta-analytic methods that do not adjust for publication bias. The first comparison is to a random-effects meta-analysis (reMA) which is regarded as the default meta-analytic method in behavioural research. The comparison of reMA and RoBMA estimates therefore quantifies the reduction in effect size obtained when researchers use RoBMA instead of the standard methodology. The second comparison is to a different version of Bayesian model-averaged meta-analysis (BMA) [31,32,34] that is identical to RoBMA apart from the fact that BMA lacks the models that adjust for publication bias; consequently, the comparison of BMA and RoBMA estimates quantifies the reduction in effect size that can be attributed solely to publication bias adjustment.

Finally, we compare the effect size adjustments due to RoBMA against the adjustments due to the methods presented by SWF. We employ the same Bayesian hierarchical models as SWF to estimate the mean publication bias adjustment, for SWF’s model selection and RoBMA separately. SWF estimated a hierarchical Bayesian model, where the effect sizes are nested within meta-analyses, which are nested within published articles. This allowed them to take into account that (i) one article often reported multiple meta-analyses and (ii) multiple estimates were generated from each meta-analysis (depending on the different adjustment methods). The models were fitted using the brms package with default weakly informative priors. In a next step, they specified four different model selection approaches based on Carter *et al.* [4]. In short, ‘model 1’ specified the presence of moderate publication bias and small effect sizes, ‘model 2’ specified the presence of high publication bias and small effect sizes, ‘model 3’ specified the presence of moderate publication bias and large effect sizes and ‘model 4’ specified the presence of high publication bias and large effect sizes. While effect size and publication bias were fixed in the four models, heterogeneity was estimated empirically from random-effects meta-analyses. SWF then selected the best-performing method in terms of RMSE and ME (mean error) for the given effect size, heterogeneity and degree of publication bias based on the results of the simulation study by Carter *et al.* [4]. For more details on their methodology, see [30, p. 8]. When analysing results from SWF’s model selection, we only estimated fixed effects when a method was selected at least 20 times. Further, we combined 3PSM and 4PSM into a single category (PSM) in line with SWF.

We performed the analysis in R [35] using the RoBMA R package [36] and additional R functions adopted from SWF and Carter *et al.* [4]. The analysis scripts and results are available at https://osf.io/7yzut/.

## 3. Results

### 3.1. Evidence for the presence of the effect

**Figure 1.**
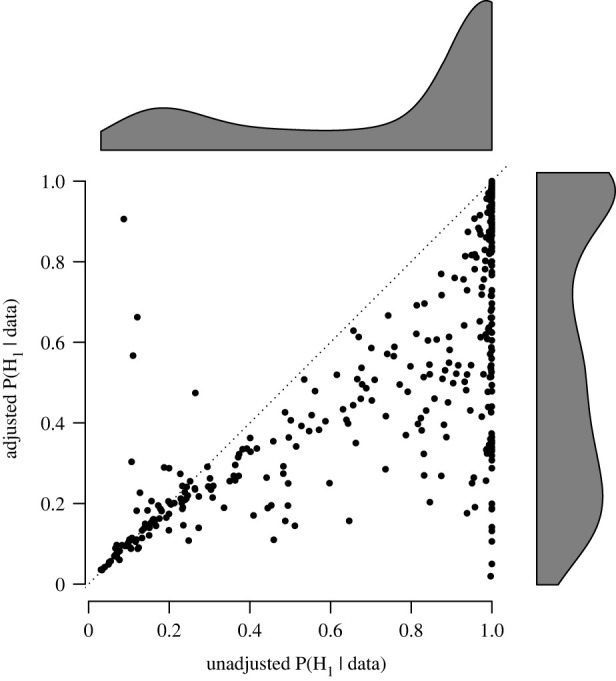
Posterior probability of the presence of the effect from the publication bias adjusted versus unadjusted models. Adjustment for publication bias reduces the probability of an effect in the majority of meta-analyses (points below the diagonal). Some meta-analyses show more evidence for an effect after publication bias adjustment. This anomaly occurs when the correction methodology adjusts a nearly zero effect size (showing evidence for the null hypothesis) further down to be slightly negative. This increases the evidence for an effect but in the *opposite* direction compared to the estimate of the original meta-analysis.

First, we used RoBMA to evaluate inflation of the posterior probability of the presence of the effect. Figure 1 shows the evidence for the presence of an effect before (*x*-axis) and after (*y*-axis) the publication bias adjustment. The dotted diagonal line highlights the points of no change in posterior probability of the alternative hypotheses due to publication bias. For many meta-analyses, the evidence for the presence of an effect is considerably lower after adjusting for publication bias, which is further exemplified by the marginal densities of the posterior probabilities on the right and top sides of the figure. Across all meta-analyses, the median posterior probability drops from 0.97, interquartile range (IRQ; 0.44, 1.00), to 0.53, IQR (0.26, 0.91), indicating considerable inflation of evidence due to publication bias. Nevertheless, for 39.2% of the meta-analyses the posterior probability for the presence of the effect did not change by more than 0.05, indicating that a notable proportion of psychology meta-analyses are relatively robust to publication bias.

Furthermore, the percentage of meta-analyses providing strong or at least moderate evidence for the alternative hypothesis (i.e. BF_10_ > 10 and BF_10_ > 3) decreased from 55.7 to 24.9% and from 64.3 to 36.9%, respectively. Interestingly, the proportion of meta-analyses providing strong or at least moderate evidence for the null hypothesis (i.e. BF_10_ < 1/10 and BF_10_ < 1/3) increased only marginally, from 4.7 to 5.2% and from 18.5 to 23.9%, respectively.^3^ Most of the change in evidence was due to the increase in the ‘undecided’ evidence category (i.e. 1/3 > BF_10_ > 3), from 17.2 to 39.2%.^4^

### 3.2. Effect size estimates

**Figure 2.**
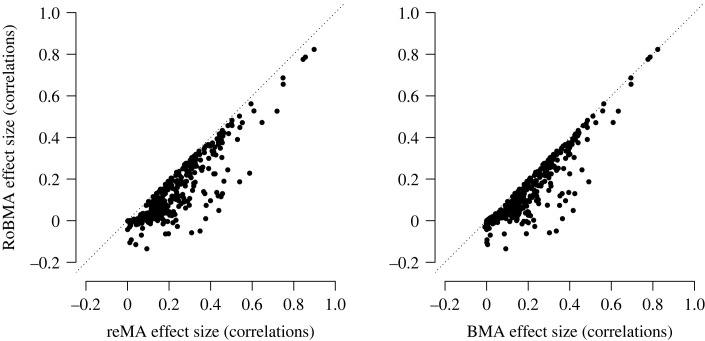
Effect size estimates from the publication bias adjusted versus unadjusted models. Model-averaged posterior mean effect size estimates based on RoBMA (*y*-axis) versus mean effect size estimates based on random-effects meta-analysis (reMA, *x*-axis, left panel) and model-averaged posterior mean effect size estimates based on BMA (*x*-axis, right panel). Adjustment for publication bias reduces effect size estimates in the majority of meta-analyses (points below the diagonal). One outlier adjusted to −0.46 (from 0.15 with BMA and 0.16 with reMA) is omitted from the display.

**Figure 3.**
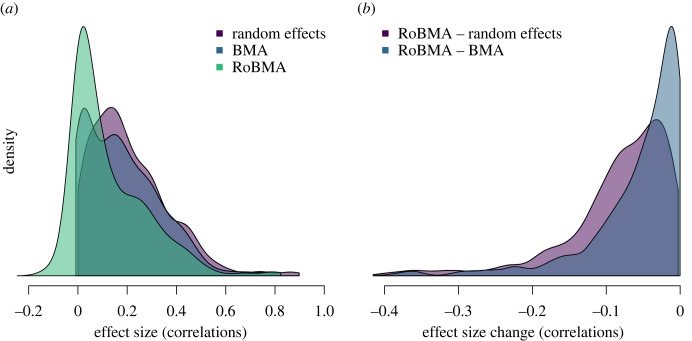
Comparison of densities of the effect size estimates from the publication bias adjusted versus unadjusted models. Densities of meta-analytic estimates under each method (*a*) and densities of differences between the unadjusted and adjusted estimates (*b*). One outlier adjusted to −0.46 is omitted from the display.

In addition to the impact on the posterior probability for the presence of the effect, we can also quantify the degree to which publication bias impacts the effect size estimates. Figures 2 and 3 show the impact of adjusting for publication bias on the meta-analytic estimates. The dotted diagonal lines in figure 2 highlight the points of no change in the effect size estimates due to publication bias. After adjusting for publication bias, many estimates are considerably smaller. Specifically, the publication bias unadjusted meta-analytic effect sizes corresponded mostly to small to medium-sized effects based on random-effects meta-analyses *r* = 0.17, IQR (0.09, 0.30), and BMA *r* = 0.15, IQR (0.04, 0.28). However, the publication bias adjustment provided by RoBMA reduced the estimates to predominantly small sized effects (i.e. *r* = 0.07, IQR (0.01, 0.22)).

Whereas the distributions of the publication bias unadjusted and adjusted effect size estimates were notably different, the distribution of differences between the estimates was highly skewed with many meta-analyses undergoing only small publication bias adjustments (figure 3*b*). The median adjustment from random-effect meta-analyses to RoBMA was *r* = −0.07, IQR (−0.11, −0.03), and the median adjustment from BMA to RoBMA was *r* = −0.03, IQR (−0.07, −0.01). Interestingly, the comparison of BMA and RoBMA, quantifying the adjustment attributable only to the publication bias adjustment part, revealed that 47.3% of meta-analytic effect size estimates are adjusted by less than *r* = 0.03,^5^ again indicating that not all meta-analytic estimates are distorted by publication bias.

van Aert *et al.* [37] argue that meta-analyses with low heterogeneity show little evidence of publication bias. To assess the impact of heterogeneity, we conducted an exploratory regression analysis predicting the effect size adjustment attributable to publication bias from heterogeneity with the unadjusted effect size estimate as a covariate (to account for the fact that meta-analyses with larger effect sizes on average show larger absolute bias and larger *τ*). We found that publication bias and heterogeneity were indeed associated, BF_10_ = 8.96 × 10^8^, *b* = −0.20, 95% CI [−0.26,−0.15]. Contrary to the conclusions of van Aert *et al.* [37], we obtain moderate evidence in favour of effect size overestimation even among homogeneous studies (i.e. no heterogeneity, tested via the coefficient for intercept), BF_10_ = 8.66, Fisher’s *z* = −0.02, 95% CI [−0.03,0.00]. However, the effect size adjustment in homogeneous meta-analyses is much smaller than the average effect size adjustment across all meta-analyses (Fisher’s *z* = −0.06, 95% CI [−0.07,−0.05]).^6^

### 3.3. Comparison to results from SWF

**Figure 4.**
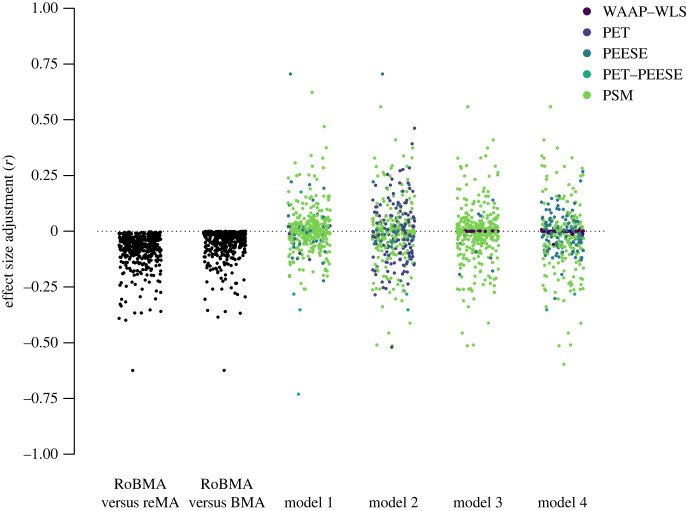
Comparison of the publication bias adjustment generated by RoBMA and the remaining methods under different models of publication bias as constructed by Sladekova *et al.* [30]. The legend colours apply to models 1–4.

We compared the effect size adjustments based on RoBMA to those based on the model selected under different assumptions about the incidence of publication bias and effect size called ‘model 1’ though ‘model 4’ by SWF. To reiterate, model 1 specified the presence of moderate publication bias and small effect sizes, model 2 specified the presence of strong publication bias and small effect sizes, model 3 specified the presence of moderate publication bias and large effect sizes and model 4 specified the presence of strong publication bias and large effect sizes. Figure 4 compares the effect size adjustments in the individual studies by RoBMA and under the different models of SWF.

The most noticeable difference between the effect size adjustments is that RoBMA did not correct any of the effect size estimates in the opposite direction. As reported before, the median effect size adjustment of RoBMA, *r* = −0.07, IQR (−0.12, − 0.04), and *r* = −0.04, IQR (−0.08, −0.01), when comparing to random-effects meta-analysis and BMA, was larger than adjustments of the other methods, 0.00 IQR (−0.04,0.03), 0.00 IQR (−0.08,0.05), 0.00 IQR (−0.04,0.03), and 0.00 IQR (−0.06,0.03) for models 1, 2, 3 and 4, respectively. The proportion of meta-analyses where RoBMA (compared to BMA) adjusted by less than *r* = 0.03 (reported earlier, 47.3%) was higher than in three out of four models; 44.8, 30.3, 50.0 and 41.9%, for models 1, 2, 3 and 4, respectively (but not for adjustment from random-effects meta-analysis to RoBMA, 23.2%). Furthermore, if we account for the direction of the effect size adjustment, SWF’s model selection procedure resulted in far fewer effect size estimates corrected downwards by *r* more than 0.03; 27.5, 37.9, 26.9 and 33.7%, for models 1, 2, 3 and 4, respectively. In other words, while RoBMA, on average, adjusted effect sizes more aggressively than other methods, it targeted the adjustment to a lower proportion of meta-analyses than the remaining methods.

**Figure 5.**
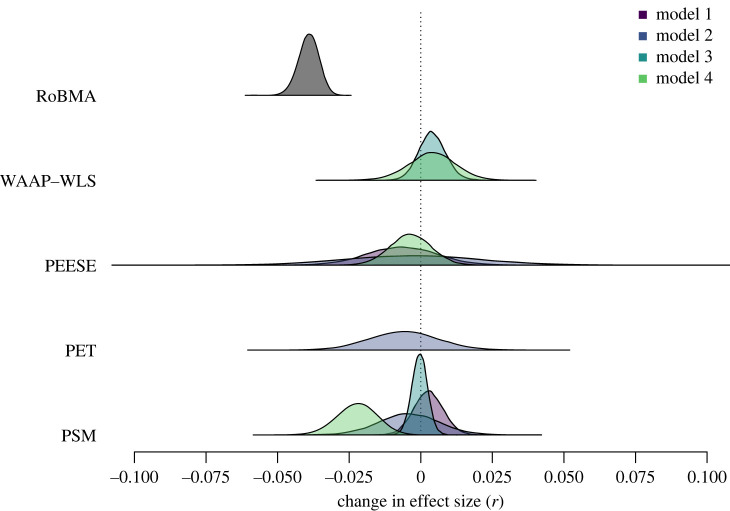
Comparison of the publication bias adjustment performed by RoBMA and the remaining methods under different models of publication bias, as constructed by SWF. We only estimated fixed effects when a method was selected at least 20 times. Specifically, WAAP-WLS was only selected for models 3 and 4 and PET only for model 2.

Finally, we estimated a three-level Bayesian model describing the effects of different publication bias adjustments with the same specification as in Sladekova *et al.* [30] (combining the 3 and 4PSM category into PSM) for RoBMA and each model separately. This three level model estimates the effect of publication bias adjustment by nesting the effect sizes within the meta-analyses (to account for the fact that estimates from the same meta-analysis may be related) and nesting meta-analyses within published articles (as published meta-analysis papers often report multiple meta-analyses). Figure 5 compares the fixed-effect estimates of the different methods under the different models. The fixed-effect estimate of the RoBMA adjustment, *β*_RoBMA_ = −0.04, 95% CI [−0.05,−0.03], is notably more negative than the adjustments of the remaining methods under model 1: *β*_PEESE_ = −0.01, 95% CI [−0.03,0.02], *β*_PSM_ = 0.00, 95% CI [−0.01,0.01], model 2: *β*_PEESE_ = 0.00, 95% CI [−0.05,0.04], *β*_PET_ = −0.01, 95% CI [−0.03,0.02], *β*_PSM_ = 0.00, 95% CI [−0.02,0.02], model 3: *β*_PSM_ = 0.00, 95% CI [−0.01,0.01], *β*_WAAP-WLS_ = 0.00, 95% CI [−0.01,0.01], or model 4: *β*_PEESE_ = 0.00, 95% CI [−0.02,0.01], *β*_PSM_ = −0.02, 95% CI [−0.04,−0.01], *β*_WAAP-WLS_ = 0.00, 95% CI [−0.01,0.02]. Table 1 further shows a comparison of the adjusted meta-analytic estimates between our study and SWF.

**Table 1.** Mean and 95% central credible intervals for the adjusted effect size estimates (*r*) of SWF’s methods under models 1–4 and the RoBMA adjusted effect size estimate from a linear three-level model.

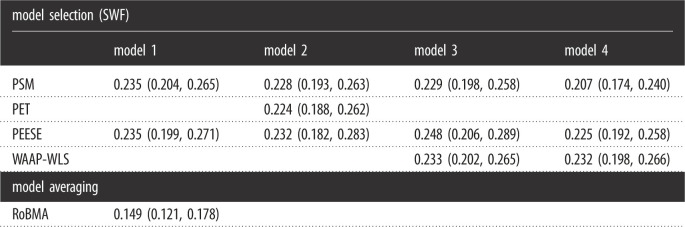

### 3.4. Sensitivity analysis for prior distribution

In addition to the normal (0,1) prior used in the main analyses, we also consider the Oosterwijk prior, Student-*t*_+_(*μ* = 0.35, *σ* = 0.10, *ν* = 3). This prior was elicited from a social psychologist (Dr Oosterwijk) to describe small effect sizes in psychology [41]. Because it is not centred at *d* = 0 but at *d* = 0.35, it reduces shrinkage towards zero and increases the ability to find evidence for small effects. Table 2 shows the results of a reanalysis with this prior. We find that both the posterior probability of an effect and the model-averaged effect size estimate is larger under the Oosterwijk prior. However, this also applies to the unadjusted BMA and not only RoBMA. Overall, we find that the overestimation is somewhat weaker both in terms of evidence for the effect as well as the size of the effect, though still considerable.

**Table 2.** Sensitivity analysis of the main results to the specification of prior distribution on the presence of the effect.

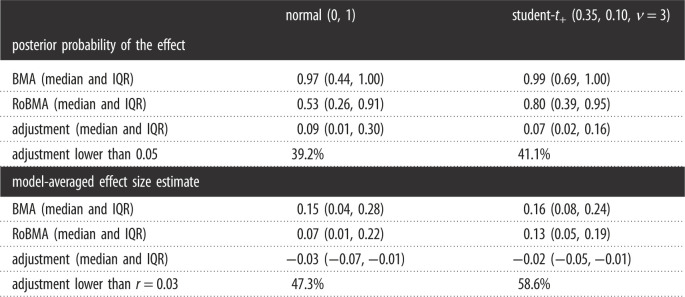

## 4. Concluding comments

It is widely accepted that different meta-analysis methods perform well under different conditions. Hence it can be risky to employ a single method to estimate the extent to which meta-analyses in general over-estimate effect sizes. SWF attempted to circumvent this complication by selecting different adjustment methods for four plausible conditions based on heterogeneity estimates indicated by a naive random-effect meta-analysis. Their article was a much needed contribution to the bias adjustment literature, being the first comprehensive review that tried to select estimators appropriate for different data-generating scenarios on an impressively large and representative dataset. Here, we outlined an alternative approach based on Bayesian model-averaging. Rather than selecting a single model for each case and assumed data generating process, our RoBMA simultaneously considers multiple models, with their contribution to the meta-analytic inference determined by their predictive accuracy.

The difference is not a point of methodological pedantry but has a considerable impact on the conclusions regarding the necessary degree of publication bias adjustment. Whereas SWF found little overestimation of effect sizes due to publication bias, similarly to van Aert *et al.* [37], and for some methods, even *larger* effects after adjustment, RoBMA often corrects more strongly and reveals the presence of notable bias. In addition, RoBMA also allowed us to assess the amount of spurious evidence, indicating that evidence for meta-analytic effect sizes is considerably weaker after publication bias is accounted for.

We point out that RoBMA has been shown to have a downward bias in the *p*-hacking simulations of Carter *et al.* [12]. Further, the truncated prior distribution on the PET and PEESE coefficients, which imposes a non-negative relationship between effect sizes and standard errors, could also reduce effect size estimates. However, the difference between the effect size correction provided by RoBMA and the remaining methods cannot be solely attributed to a downward bias of RoBMA. First, RoBMA did not adjust effect sizes downward in many of the analysed meta-analyses. Second, in appendix E of Bartoš *et al.* [11], we applied RoBMA to 28 meta-analyses from Many Labs 2 [42], a multi-laboratory Registered Replication Report, where we know that publication bias is absent. Therefore, if a method still detects publication bias or notably corrects the estimate downwards, this is likely indicative of bias. When we applied RoBMA to the Many Labs 2 dataset we found no notable downward bias, unlike other publication bias adjustment methods.

Our analysis shows that it is important to employ multi-model methods when adjusting for publication bias, as model selection is problematic in the absence of strong knowledge about the data generating process. Our extension of the SWF work suggests that the effects of publication bias are more deleterious than previously estimated. However, it remains the case that for a sizeable proportion of studies, the correction is relatively modest. The considerable overestimation of effect sizes and evidence for the effect highlights the importance of using appropriate bias correction methods and the imperative to adopt publishing formats that are robust to publication bias, such as Registered Reports [15].

## Data Availability

The data and R scripts for performing the analyses are openly available on OSF at https://osf.io/7yzut/.

## Footnotes

1 The type and degree of publication bias as well as the true effect size is generally unknown, a problem which SWF sidestepped by calculating four possible models of publication bias and effect size. Moreover, the random-effects heterogeneity estimates that are required to select the best method depend on the degree and type of publication bias [6,7].

2 Use of the Fisher *z* transformation did necessitate the removal of 51 reported correlation coefficients equal to 1 from 4 meta-analyses. This procedure might lead to somewhat reduced estimates. However, these estimates would also pose challenges in the standard meta-analytic framework as the associated standard errors would be 0. Further, as a perfectly proportional relationship between two variables in psychology seems unlikely, these coefficients may have arisen from error in coding or in the primary study. Finally, only a very small proportion of all estimates are affected by this removal. For these reasons, we do not regard overestimation due to this removal as a practical concern.

3 This can be partly explained by the difficulty in finding evidence for the null using priors centred on zero as in RoBMA (and most other applications of Bayesian testing). We still chose to use these priors for the main analysis, as they have other desirable properties and they have been evaluated extensively in applied examples and simulation studies [11].

4 While it is impossible to evaluate the change of evidence with the frequentist methods employed by SWF (as *p*-values are not a direct measure of evidence), we summarize the change in statistical significance with *α* = 0.05, where out of the original 294 statistically significant meta-analytic effect size estimates, 77.2, 62.2, 75.0 and 71.3% remained statistically significant for Models 1, 2, 3 and 4, respectively.

5 Other possible ‘thresholds’ would result in 24.1% with *r* = 0.01, 37.7% with *r* = 0.02, 54.4% with *r* = 0.04 and 63.5% with *r* = 0.05.

6 We calculated estimates and CIs using a standard linear model in R and corresponding Bayes factors using a normal approximation [e.g. 38–40] specifying centred normal prior distributions with a standard deviation of 0.6. This prior corresponds to a standard deviation of 0.3 on Cohen’s *d* scale, which tests for small effects.

## References

[1] Rosenthal R, Gaito J. 1964 Further evidence for the cliff effect in interpretation of levels of significance. Psychol. Rep. **15**, 570. (10.2466/pr0.1964.15.2.570)

[2] Vevea JL, Hedges LV. 1995 A general linear model for estimating effect size in the presence of publication bias. Psychometrika **60**, 419-435. (10.1007/BF02294384)

[3] Borenstein M, Hedges LV, Higgins JP, Rothstein HR. 2009 Introduction to meta-analysis. Hoboken, NJ: John Wiley & Sons.

[4] Carter EC, Schönbrodt FD, Gervais WM, Hilgard J. 2019 Correcting for bias in psychology: a comparison of meta-analytic methods. Adv. Methods Pract. Psychol. Sci. **2**, 115-144. (10.1177/2515245919847196)

[5] Heller J. 1961 Catch-22. Hoboken, NJ: Simon and Schuster.

[6] Augusteijn HE, van Aert R, van Assen MA. 2019 The effect of publication bias on the Q test and assessment of heterogeneity. Psychol. Methods **24**, 116-134. (10.1037/met0000197) 30489099

[7] Hönekopp J, Linden AH. 2022 Heterogeneity estimates in a biased world. PLoS ONE **17**, 1-21. (10.1371/journal.pone.0262809) PMC8812955 35113897

[8] Stanley TD, Doucouliagos H, Ioannidis JPA, Carter EC. 2021 Detecting publication selection bias through excess statistical significance. Res. Synth. Methods **12**, 776-795. (10.1002/jrsm.1512) 34196473

[9] Hinne M, Gronau QF, Wagenmakers EJ. 2020 A conceptual introduction to Bayesian model averaging. Adv. Methods Pract. Psychol. Sci. **3**, 200-215. (10.1177/2515245919898657)

[10] Hoeting JA, Madigan D, Raftery AE, Volinsky CT. 1999 Bayesian model averaging: a tutorial. Stat. Sci. **14**, 382-401. (10.1214/ss/1009212519)

[11] Bartoš F, Maier M, Wagenmakers EJ, Doucouliagos H, Stanley TD. 2022a Robust Bayesian meta-analysis: model-averaging across complementary publication bias adjustment methods. Res. Synth. Methods **14**, 99-116. (10.1002/jrsm.1594) 35869696 PMC10087723

[12] Bartoš F, Maier M, Quintana D, Wagenmakers EJ. 2022b Adjusting for publication bias in JASP and R: selection models, PET-PEESE, and robust Bayesian meta-analysis. Adv. Methods Pract. Psychol. Sci. **5**, 1-19. (10.1177/25152459221109259)

[13] Maier M, Bartoš F, Wagenmakers EJ. 2022 Robust Bayesian meta-analysis: addressing publication bias with model-averaging. Psychol. Methods **28**, 107-122. (10.1037/met0000405) 35588075

[14] Kvarven A, Strømland E, Johannesson M. 2020 Comparing meta-analyses and preregistered multiple-laboratory replication projects. Nat. Hum. Behav. **4**, 423-434. (10.1038/s41562-019-0787-z) 31873200

[15] Chambers CD, Dienes Z, McIntosh RD, Rotshtein P, Willmes K. 2015 Registered reports: realigning incentives in scientific publishing. Cortex **66**, A1-A2. (10.1016/j.cortex.2015.03.022) 25892410

[16] Chambers CD. 2013 Registered reports: a new publishing initiative at Cortex. Cortex **49**, 609-610. (10.1016/j.cortex.2012.12.016) 23347556

[17] Lewis M, Mathur MB, VanderWeele TJ, Frank MC. 2020 The puzzling relationship between multi-lab replications and meta-analyses of the rest of the literature. *R. Soc. Open Sci.* **9**, 211499. (10.1098/rsos.211499) PMC8864345 35223059

[18] Jeffreys H. 1961 Theory of probability, 3rd edn. Oxford, UK: Oxford University Press.

[19] Jeffreys H. 1973 Scientific inference, 3rd edn. Cambridge, UK: Cambridge University Press.

[20] Wagenmakers EJ, Morey RD, Lee MD. 2016 Bayesian benefits for the pragmatic researcher. Curr. Direct. Psychol. Sci. **25**, 169-176. (10.1177/0963721416643289)

[21] Wagenmakers EJ. 2007 A practical solution to the pervasive problems of *p* values. Psychon. Bull. Rev. **14**, 779-804. (10.3758/BF03194105) 18087943

[22] Gronau QF, Wagenmakers EJ. 2018 Bayesian evidence accumulation in experimental mathematics: a case study of four irrational numbers. Exp. Math. **27**, 277-286. (10.1080/10586458.2016.1256006)

[23] van Erp S, Verhagen J, Grasman RP, Wagenmakers EJ. 2017 Estimates of between-study heterogeneity for 705 meta-analyses reported in Psychological Bulletin from 1990–2013. J. Open Psychol. Data **5**, 4. (10.5334/jopd.33)

[24] Maier M, VanderWeele TJ, Mathur MB. 2022 Using selection models to assess sensitivity to publication bias: a tutorial and call for more routine use. Campbell Syst. Rev. **18**, e1256. (10.1002/cl2.1256) 36909879 PMC9247867

[25] Larose DT, Dey DK. 1998 Modeling publication bias using weighted distributions in a Bayesian framework. Comput. Stat. Data Anal. **26**, 279-302. (10.1016/S0167-9473(97)00039-X)

[26] Stanley TD, Doucouliagos H, Ioannidis JP. 2017 Finding the power to reduce publication bias. Stat. Med. **36**, 1580-1598. (10.1002/sim.7228) 28127782

[27] Hong S, Reed WR. 2020 Using Monte Carlo experiments to select meta-analytic estimators. Res. Synth. Methods **12**, 192-215. (10.1002/jrsm.1467) 33150663 PMC8074967

[28] Alinaghi N, Reed WR. 2018 Meta-analysis and publication bias: How well does the FAT-PET-PEESE procedure work? Res. Synth. Methods **9**, 285-311. (10.1002/jrsm.1298) 29532634

[29] Bom PR, Rachinger H. 2019 A kinked meta-regression model for publication bias correction. Res. Synth. Methods **10**, 497-514. (10.1002/jrsm.1352) 31039283

[30] Sladekova M, Webb LEA, Field AP. 2022 Estimating the change in meta-analytic effect size estimates after the application of publication bias adjustment methods. Psychol. Methods **28**, 664-686. (10.1037/met0000470) 35446048

[31] Gronau QF, van Erp S, Heck DW, Cesario J, Jonas KJ, Wagenmakers EJ. 2017 A Bayesian model-averaged meta-analysis of the power pose effect with informed and default priors: the case of felt power. Compr. Results Soc. Psychol. **2**, 123-138. (10.1080/23743603.2017.1326760)

[32] Bartoš F, Gronau QF, Timmers B, Otte WM, Ly A, Wagenmakers EJ. 2021 Bayesian model-averaged meta-analysis in medicine. Stat. Med. **40**, 6743-6761. (10.1002/sim.9170) 34705280 PMC9298250

[33] Lee MD, Wagenmakers EJ. 2013 Bayesian cognitive modeling: a practical course. New York, NY: Cambridge University Press.

[34] Gronau QF, Heck DW, Berkhout SW, Haaf JM, Wagenmakers EJ. 2021 A primer on Bayesian model-averaged meta-analysis. Adv. Methods Pract. Psychol. Sci. **4**, 1-19. (10.1177/25152459211031256) PMC10991068 37099263

[35] R Core Team. 2021 *R: a language and environment for statistical computing*. R Foundation for Statistical Computing Vienna, Austria.

[36] Bartoš F, Maier M. 2020 RoBMA: an R package for robust Bayesian meta-analyses. R package version 2.1.1.

[37] van Aert RC, Wicherts JM, Van Assen MA. 2019 Publication bias examined in meta-analyses from psychology and medicine: a meta-meta-analysis. PLoS ONE **14**, e0215052. (10.1371/journal.pone.0215052) 30978228 PMC6461282

[38] Bartoš F, Wagenmakers EJ. 2022 Fast and accurate approximation to informed Bayes factors for focal parameters. (http://arxiv.org/abs/2203.01435)

[39] Spiegelhalter DJ, Abrams KR, Myles JP. 2004 Bayesian approaches to clinical trials and health-care evaluation. Chichester, UK: John Wiley & Sons.

[40] Dienes Z. 2014 Using Bayes to get the most out of non-significant results. Front. Psychol. **5**, 781. (10.3389/fpsyg.2014.00781) 25120503 PMC4114196

[41] Gronau QF, Ly A, Wagenmakers EJ. 2020 Informed Bayesian *t*-tests. Am. Stat. **74**, 137-143. (10.1080/00031305.2018.1562983)

[42] Klein RA et al. 2018 Many Labs 2: investigating variation in replicability across samples and settings. Adv. Methods Pract. Psychol. Sci. **1**, 443-490. (10.1177/2515245918810225)

